# A literature review: progress in the study of plastic bronchitis

**DOI:** 10.3389/fped.2026.1759935

**Published:** 2026-05-04

**Authors:** Huihan Li, Chi Han, Danchen Wu, Yuanyuan Zhang

**Affiliations:** Department of Pulmonology, Children’s Hospital, Zhejiang University School of Medicine, National Clinical Research Center for Child Health, Hangzhou, China

**Keywords:** child, fontan, mycoplasma pneumoniae, pathogenesis, plastic bronchitis, virus

## Abstract

This literature review aims to synthesize current evidence on plastic bronchitis (PB), a disease characterized by varying degrees of branching cast formation in the airways. The rationale for undertaking this review stems from the increasing recognition of PB as a potentially life-threatening condition in pediatric populations, coupled with significant recent advances in diagnostic and therapeutic approaches. By systematically searching and analyzing historical literature related to PB from multiple electronic databases, this review examines the current status of PB studies across five critical domains: risk factors, pathology and pathogenesis, clinical features and diagnosis, treatment, and prognosis. Particular emphasis is placed on recent advances in diagnostic imaging, interventional bronchoscopy techniques, and emerging pharmacological therapies. This review provides clinically relevant guidance to enhance awareness and improve management strategies for this rare but serious condition among pediatric practitioners.

## 1 Introduction

Plastic bronchitis (PB), also known as tubular bronchitis and fibrinous bronchitis (1), is an airway obstructive disease characterized by gelatinous branching cast formation. These casts can cause hypoxemia, severe respiratory distress, and even death, with a prevalence of approximately 6.8 per 100,000 in pediatric populations (2–4). PB is commonly associated with congenital heart disease and primary pulmonary disease (5).

The clinical presentation of PB is often insidious at onset but can progress rapidly, becoming life-threatening if airway obstruction is not relieved promptly. Although the number of reported PB cases has gradually increased with the widespread adoption of bronchoscopy in pediatric practice, awareness of this condition among general pediatricians remains insufficient. This review aims to synthesize the current evidence on risk factors, pathology, pathogenesis, clinical features, diagnosis, management, and prognosis of PB, with particular attention to recent advances in diagnostic imaging, interventional bronchoscopy, and emerging pharmacotherapies, in order to provide clinicians with actionable guidance for patient care.

## 2 Methodology

### 2.1 Search strategy

A comprehensive literature search was conducted across PubMed/MEDLINE, Embase, and Web of Science databases from their inception through March 2025. The search employed the following keywords and Medical Subject Headings (MeSH) terms, combined using Boolean operators: (“plastic bronchitis” OR “bronchial casts” OR “fibrinous bronchitis” OR “tubular bronchitis” OR “cast bronchitis”) AND (“pediatric” OR “children” OR “infant” OR “adolescent”). Searches were further refined using subject filters for etiology, pathogenesis, diagnosis, treatment, and prognosis. Additionally, the reference lists of all included articles and relevant recent systematic reviews were manually screened to identify any additional relevant publications.

### 2.2 Selection criteria

Inclusion criteria were: (1) original research articles (randomized controlled trials, cohort studies, case-control studies, cross-sectional studies), case series (≥3 patients), and systematic reviews/meta-analyses focusing on plastic bronchitis; (2) studies involving pediatric populations (age < 18 years) or studies with pediatric subgroup data; (3) articles published in English or those with available English-language translations; (4) studies reporting clinical characteristics, diagnostic methods, treatment approaches, or outcomes of plastic bronchitis.

Exclusion criteria were: (1) single case reports of adult patients without pediatric relevance; (2) conference abstracts and posters without full-text peer-reviewed availability; (3) duplicate publications reporting the same patient cohort; (4) editorials, commentaries, and letters without original data; (5) studies written in languages other than English without available translations.

### 2.3 Data extraction and evidence assessment

Data were extracted regarding study design, patient demographics, underlying conditions, cast characteristics, diagnostic modalities, treatment strategies, and clinical outcomes. The quality of evidence for each topic area was assessed qualitatively based on study design hierarchy as follows: Level I (systematic reviews and meta-analyses), Level II (randomized controlled trials), Level III (prospective cohort studies), Level IV (retrospective cohort and case-control studies), and Level V (case series and case reports). This grading system was adapted from the Oxford Centre for Evidence-Based Medicine (OCEBM) levels of evidence. Throughout the review, the level of evidence supporting key statements is explicitly indicated.

### 2.4 Search results

A total of 217 articles were initially identified through database searches, and 18 additional articles were identified through manual screening of reference lists. After removal of 73 duplicates, 162 articles underwent title and abstract screening, of which 89 were selected for full-text review. Ultimately, 58 articles met the inclusion criteria and were incorporated into this review.

## 3 Risk factors

### 3.1 Cardiac-associated risk factors

PB is most commonly associated with underlying cardiovascular anomalies. Children with complex congenital heart disease—particularly those with tricuspid atresia, pulmonary atresia, or hypoplastic left heart syndrome (6)—who undergo staged palliative surgery such as the Fontan, Glenn, or Blalock-Taussig operations, are at higher risk (7–10). The prevalence of PB following staged palliative surgery for complex congenital heart disease ranges from 4 to 14% (Level IV). The pathogenesis of PB in this setting may involve impaired lymphatic drainage secondary to the elevated central venous pressure and morphologic changes in the thoracic duct (9, 11–14). In a case-control study by Schumacher et al. (8), the development of PB was significantly associated with a longer duration of Fontan circulation, prolonged chest tube retention, postoperative protein-losing enteropathy, and postoperative ascites. However, the occurrence, severity, and frequency of PB episodes vary considerably among patients with congenital heart disease.

### 3.2 Lymphatic anomalies

PB may also be associated with non-cardiac thoracic lymphatic abnormalities, which are typically secondary to genetic syndromes such as Noonan syndrome, Turner syndrome, and congenital lymphadenopathy (15, 16) (Level V). These conditions highlight the role of lymphatic system dysregulation in the pathophysiology of PB, independent of cardiac surgery.

### 3.3 Infectious risk factors

Pulmonary infections represent another major category of risk factors for PB. A systematic review to date identified infection as the predominant etiology (74%) (17) (Level I). Among these, influenza virus infection—particularly influenza A— is the most frequently reported viral trigger (18–20) (Level IV-V). The pathophysiological process of influenza-associated PB may involve eosinophil activation and the release of extracellular traps (ETs), which promote mucus plug formation and facilitate Charcot-Leyden crystal generation through galactose lectin-10 crystallization (18, 21, 22). Other viruses implicated in PB include adenovirus (23), bocavirus (24), parainfluenza virus (25), respiratory syncytial virus (26), and coronavirus disease 2019 (COVID-19) (27).

*Mycoplasma pneumoniae* (MP) infection is an increasingly recognized cause of PB, particularly in East Asian populations (20, 21). Case-control studies have identified prolonged fever, marked inflammatory response, and immune dysfunction as risk factors for MP-associated PB include (28, 29) (Level IV). He et al. (30) recently conducted a multicenter cohort study to date, encompassing 180 pediatric PB patients from China, revealed that viral pathogens—particularly human bocavirus—predominated in the youngest age group (0–3 years), whereas MP was the dominant pathogen in children aged >3 years.(Level IV).

### 3.4 Other risk factors

Additional less common associations include sickle cell anemia acute chest syndrome (SCACS), allergic bronchopulmonary aspergillosis (ABPA), hypersecretory asthma, and chronic obstructive pulmonary disease (COPD) (1, 31, 32) (Level V). These association are primarily based on isolated case reports and small case series, and the causal mechanisms remain poorly understood (Table 1).

**Table 1:** Classification of risk factors for pediatric plastic bronchitis.

Category	Specific Risk Factors	Prevalence	Evidence Level
Cardiac	Post-Fontan/Blalock-Taussig/Glenn procedures; tricuspid atresia; pulmonary atresia; hypoplastic left heart syndrome (7–10)	4–14% post-cardiac surgery (5)	Level IV
Lymphatic	Noonan syndrome; Turner syndrome; congenital lymphatic disorders (15, 16)	Rare	Level V
Viral Infections	Influenza A/B (18–20); adenovirus (23); RSV (26); parainfluenza (25); bocavirus (24); COVID-19 (27)	Dominant in infants (0–3 yrs) (30)	Level IV-V
Bacterial Infections	MP (28, 29)	Dominant in children >3 yrs (30)	Level IV
Others	SCACS; ABPA; COPD; asthma; atopic disease (1, 31, 32)	Rare	Level V

## 4 Pathology and pathogenesis

### 4.1 Cast classification

In 1997 Seear et al. (33) proposed a histopathological classification that remains widely used: type I inflammatory casts, composed predominantly of fibrin with neutrophil or eosinophil infiltration, and type II acellular casts, composed mainly of mucin with little or no cellular infiltration (Level IV). Type I casts are typically associated with inflammatory and infectious pulmonary conditions, whereas type II casts are more commonly linked to structural heart disease, particularly single ventricle physiology after Fontan procedure. However, this correlation is not absolute; children with structural heart disease may develop fibrin-predominant casts, and those with asthma or atopic disease may produce mucus-predominant casts with varying degrees of cellular infiltration (5, 34) (Table 2).

**Table 2:** Comparison of seear cast classification systems in plastic bronchitis.

Feature	Type I/infalmmatory	Type II/acellular
Composition	Fibrin with neutrophil/eosinophil infiltrate	Mucin with minimal cellular infiltration (33)
Associated conditions	Infections (viral, MP); asthma; atopic disease	Post-Fontan surgery; congenital heart disease; lymphatic disorders (5, 33)
Pathogenesis	Inflammatory cascade; ET formation; cytokine-mediated (21, 29)	Lymphatic leak; mucin cross-linking; elevated venous pressure (9, 11)

More recently, a lymphatic-based classification has emerged, particularly for post-Fontan PB, which categorizes disease based on lymphatic flow abnormalities identified by dynamic contrast MR lymphangiography (DCMRL) (11, 12). This classification identifies patterns of abnormal lymphatic perfusion, such as central lymphatic channel dilation, retrograde lymphatic flow, and lymphatic-to-airway fistulas. While this classification provides valuable anatomical guidance for interventional planning, it is limited to cardiac-associated PB and requires specialized imaging that is not universally available. The Seear histopathological classification remains more broadly applicable but lacks specificity for guiding treatment. Ideally, an integrated approach combining both histopathological and lymphatic imaging data would provide the most comprehensive framework for clinical decision-making, although such a unified classification has yet to be formally validated.

### 4.2 Pathogenic mechanisms

The pathogenesis of cast formation is multifactorial and incompletely understood. In cardiac-associated PB, the Fontan procedure establishes a direct connection between the systemic veins and the pulmonary circulation, bypassing the right ventricle. Dori et al. (11) demonstrated through magnetic resonance imaging (MRI) that patients with post-Fontan PB exhibit specific abnormalities of the thoracic lymphatics, including thoracic duct dilation and abnormal lymphatic perfusion patterns (Level IV). Selective embolization of these abnormal lymphatic channels has been shown to reduce or prevent cast formation (12) (Level IV). The proposed mechanism involves elevated central venous pressure compromising tracheal mucosal integrity, impeding lymphatic drainage, and promoting the development of lymphoalveolar fistulas through which proteinaceous material leaks into the airway lumen (31).

In infection-associated PB, cast formation appears to involve eosinophil activation and extracellular trap (ET) release, particularly in influenza-associated cases (34). In MP-associated PB, neutrophil-mediated inflammation and dysregulated immune responses are thought to contribute to cast formation (35, 36) (Level IV). Elevated expression levels of MUC5AC, MUC5B, and layilin in bronchoalveolar lavage fluid (BALF) by MP activating the STAT6-STAT3 and epidermal growth factor receptor signal pathways were reported to be predictive factors in the development of MP-associated PB and be relevant to the severity of illness (37), providing with underlying mechanisms of PB formation and potential treatment target (Level IV). Additionally, mucin cross-linking—where adjacent mucin polymers form abnormal intermolecular bridges distinct from those in normal airway mucus—has been identified as a key factor in cast stability and tenacity (5) (Level V).

## 5 Clinical features and diagnosis

### 5.1 Clinical presentation

PB typically presents with cough, wheezing, dyspnea, and chest pain, accompanied by signs of varying degrees of airway obstruction signs, including hypoxemia and potential severe respiratory distress (2) (Level IV). The expectoration of large, branched, brown rubbery casts is a pathognomonic feature but is not present in all cases. Physical chest examination may reveal diminished breath sounds or localized wheezing (2). In young children who cannot expectorate, the diagnosis may be delayed, making a high index of clinical suspicion essential.

### 5.2 Diagnostic imaging

Diagnostic imaging has progressed from merely identifying complications to uncovering the underlying pathophysiology of PB. Chext x-ray (CXR) typically serves as the initial screening tool. It may show localized pulmonary atelectasis with compensatory hyperventilation of adjacent lung segments, air bronchograms, or peribronchial thickening (38) (Level IV). However, CXR findings are frequently non-specific and may be misinterpreted as pneumonia, atelectasis from other causes, or foreign body aspiration.

Computed tomography (CT) provides superior anatomical detail compared to CXR. CT may demonstrate bronchial wall thickening, mucoid impaction, and air trapping. High-resolution CT (HRCT) is particularly valuable for identifying complications such as pulmonary thromboembolism, which has been increasingly recognized in MP-associated PB (29, 39). O'Leary et al. (40) conducted a retrospective study of 44 patients with PB and found that ground-glass opacities (GGO) were present in 91% of lymphatic PB patients compared with only 33% of non-lymphatic patients (*P* < 0.001, OR = 21, 95% CI: 3.8–159.7), with multifocal distribution predominantly in the lower lobes (Level IV). This finding suggests that the presence of diffuse GGO on chest CT should prompt further lymphatic workup.

MRI has emerged as a valuable tool for evaluating lymphatic abnormalities in PB, particularly in post-Fontan patients. DCMRL enables real-time visualization of lymphatic flow patterns and identification of abnormal lymphatic channels that may contribute to cast formation (11, 12). This non-invasive imaging modality has revolutionized the understanding of lymphatic involvement in PB and guides interventional planning. Additionally, Hao et al. (41) compared CT lymphangiography (CTL) with MR lymphangiography (MRL) in 31 patients with lymphatic PB and found that CTL was superior for detecting pulmonary parenchymal abnormalities including lobular septal thickening, pulmonary nodules, and airway stenosis, whereas MRL was better for evaluating central lymphatic structures such as the thoracic duct and right lymphatic duct. Combined imaging provided complementary information.

### 5.3 Bronchoscopy

Flexible bronchoscopy is the cornerstone for definitive diagnosis of PB when cast expectoration has not occurred (5). It allows direct visualization and extraction of endobronchial casts, provide rapid relief of airway obstruction, and enables collection of bronchoalveolar lavage (BAL) specimens for dianostic analysis (25, 42–44). Pathogen detection should be performed through bronchoalveolar (BAL) fluid analysis, including bacterial culture, viral polymerase chain reaction (PCR) testing, and MP-specific antibody or PCR detection, to identify the potential cause of infection-related PB and guide treatment of primary disease. Cast material should be submitted for histopathological examination to determine the Seear classification, which can guide subsequent management (Table 3).

**Table 3:** Dianostic approach for pediatric plastic bronchitis.

Modality	Key Findings	Clinical Utility
CXR	Atelectasis; peribronchial thickening; air bronchograms	Initial screening; low specificity (38)
CT/HRCT	Tree-in-bud pattern; mucoid impaction; air trapping; GGO	Anatomical detail; complication detection (29, 39)
DCMRL	Abnormal lymphatic channels; lymphatic reflux; fistulas	Lymphatic assessment; guides embolization (11, 12)
Flexible bronchoscopy	Visualized casts; airway inflammation	Definitive diagnosis; therapeutic removal (42, 43)
BAL analysis	Pathogen identification; inflammatory cell profiling	Etiological diagnosis; treatment guidance
Cast histopathology	Fibrin vs. mucin; cellular infiltrate classification	Seear typing; treatment stratification

## 6 Management

The overall goals of PB management are: (1) relief of acute airway obstruction, (2) identification and treatment of underlying conditions, and (3) prevention of cast recurrence. Management strategies differ substantially between cardiac-associated and non-cardiac (infection/inflammatory-associated) PB.

### 6.1 Acute management

#### 6.1.1 Bronchoscopic intervention

The first-line intervention for acute PB is prompt airway clearance through bronchoscopy. Both rigid and flexible bronchoscopy can be used, with rigid bronchoscopy preferred in severe cases requiring rapid airway stabilization and in younger children (45) (Level IV-V). Serial bronchoscopic interventions are often necessary to achieve adequate control of symptoms and complete evacuation of endobronchial casts and debris. Multiple sessions—ranging from 2 to over 10 procedures—may be necessary, particularly in severe cases with extensive cast burden (44, 46).

Therapeutic BAL plays a crucial role in both diagnosis and treatment. Therapeutic BAL helps to liquefy and remove viscous secretions, dilute inflammatory mediators, and improve mucociliary clearance (42). Various cast extraction methods have been described, including direct forceps extraction, suctioning with large-bore catheters, and fragmenting casts with saline lavage (42, 44, 46). Cryotherapy via flexible bronchoscopy has also been reported as an effective adjunctive technique for refractory cases (43). The choice of technique depends on cast consistency, location, and patient tolerance (Level V).

#### 6.1.2 Supportive therapies

For patients with impending or established respiratory failure who cannot be managed with bronchoscopy alone, high-frequency jet ventilation has been reported as a useful adjunctive therapy (45) (Level V). Extracorporeal membrane oxygenation (ECMO) may be considered as a rescue therapy for children who progress to refractory respiratory or circulatory failure, although outcomes in this population remain guarded (47) (Level V).

### 6.2 Management of cardiac-associated PB

#### 6.2.1 Medical therapy

Aerosolized heparin is a commonly used first-line therapy for cardiac-associated PB. Heparin is thought to act through anti-inflammatory effects and fibrinolysis mediated by antithrombin III activation (48). A typical regimen involves aerosolized unfractionated heparin at a dose of 5,000–10,000 units diluted in 3–5 mL of normal saline, administered 2–4 times daily via nebulization (48) (Level V). Heparin is generally better tolerated than inhaled tissue plasminogen activator (tPA), causing less airway irritation (5).

Aerosolized tPA represents a more aggressive fibrinolytic approach. The PLATyPuS trial (49), a phase II multicenter open-label study (NCT02315898), has provided prospective safety data on nebulized tPA for pediatric PB (Level II). In this study, 8 hospitalized patients with acute PB exacerbations received nebulized tPA at 5 mg per dose via jet nebulizer every 6 h for 72 h. Key safety findings included: no clinically significant disruption of systemic coagulation parameters at any time point; and oxygen saturation showed statistically significant improvement following treatment, though without clinically meaningful lung function gains. Common adverse effects in the broader literature include transient hemoptysis and bronchospasm (50). tPA is generally reserved for patients who fail to respond to heparin therapy or who present with particularly tenacious fibrin-rich casts.

#### 6.2.2 Lymphatic intervention

When medical therapy fails, interventional lymphatic management should be considered. DCMRL and intra-nodal lymphangiography can identify abnormal lymphatic vessel morphology and pathological lymphatic reflux patterns (11, 12). Under imaging guidance, selective embolization of abnormal lymphatic channels has been demonstrated to effectively improve PB symptoms and reduce cast recurrence (12) (Level IV). Thoracic duct ligation may also be considered in selected cases. In the most severe and refractory cases, Fontan conversion or heart/lung transplantation may provide definitive resolution (51, 52) (Level V).

### 6.3 Management of infection/inflammatory-associated PB

#### 6.3.1 Anti-microbial therapy

For infection-triggered PB, prompt identification and treatment of the causative pathogen are essential. For MP-associated PB, macrolide antibiotics (azithromycin 10 mg/kg/day or clarithromycin 15 mg/kg/day) should be initiated, with the recognition that refractory cases may require additional immunomodulatory therapy (28, 29) (Level IV).

#### 6.3.2 Anti-inflammatory and immunomodulatory therapy

Macrolides possess immunomodulatory properties beyond their antimicrobial effects. Low-dose, long-term macrolide therapy (e.g., azithromycin 5 mg/kg 3 times per week) may reduce mucin production and cast recurrence by inhibiting extracellular signal-regulated kinase 1/2 (ERK1/2) phosphorylation and nuclear factor kappa B (NF-κB) activation (53) (Level V).

Corticosteroids are frequently used in eosinophilic and allergic PB. Systemic corticosteroids can reduce airway inflammation and cast formation (17) (Level V). However, no PB-specific dosing guidelines exist for systemic corticosteroids, and current regimens are extrapolated from broader pediatric respiratory evidence.

Biological agents have emerged as promising therapies for refractory eosinophilic PB. Mepolizumab, an anti-interleukin-5 monoclonal antibody administered subcutaneously at 12 mg (for children ≥6 years) every 4 weeks, has shown efficacy in reducing cast recurrence in small case series (54) (Level V). Dupilumab (anti-IL-4/IL-13), administered subcutaneously at weight-based dosing (300 mg every 2 weeks for children ≥6 years), has also been reported to be effective in refractory cases (55) (Level V). These biologic therapies represent a targeted approach for patients with evidence of Th2-mediated inflammation who fail conventional therapy (Table 4).

**Table 4:** Comparison of treatment approaches: cardiac-associated vs. non-cardiac PB.

Aspect	Cardiac-associated PB	Infection/infalmmatory PB
Primary mechanism	Lymphatic leak; mucin-predominant casts	Inflammation; fibrin-predominant casts
First-line therapy	Aerosolized heparin (48); bronchoscopic clearance (44, 46)	Targeted antimicrobial therapy (28, 29); bronchoscopic clearance
Second-line therapy	Aerosolized tPA (49); lymphatic embolization (49)	Systemic/inhaled corticosteroids (17); macrolides (53)
Third-line therapy	Fontan revision; transplantation (51, 52)	Biologic agents (mepolizumab; dupilumab) (54, 55)
Prevention focus	Lymphatic flow optimization; cardiac hemodynamics	Infection prevention; inflammation control; trigger avoidance

## 7 Prognosis

The prognosis of PB is closely linked to the underlying primary disease and cast type.

### 7.1 Cardiac-associated PB

For patients with complex congenital heart disease who have undergone surgical palliation, PB carries a high mortality rate, at approximately 14% to 50% (31) (Level IV). A single-center follow-up study of reported that freedom from death or transplantation at 5, 10, and 15 years after diagnosis of protein-losing enteropathy (PLE)—a closely related Fontan complication— or PB was 70% (95% CI, 58–85), 65% (95% CI, 51–83), and 43% (95% CI, 26–73), respectively (56) (Level IV). It should be noted that this study combined PLE and PB outcomes, and the prognosis specifically for isolated PB may differ. Successful lymphatic embolization has been associated with improved outcomes in select patients with post-Fontan PB (12).

### 7.2 Infection/inflammatory-associated PB

For PB associated with asthma or atopic disease, the reported mortality rate ranges from 6% to 50%, depending on the severity and timeliness of intervention (5) (Level V). In MP-associated PB, early bronchoscopic intervention combined with appropriate antimicrobial therapy is generally associated with favorable outcomes, although refractory cases with extensive cast burden may require prolonged treatment and carry a worse prognosis (28, 29).

### 7.3 General prognostic considerations

Regardless of the underlying etiology, mortality increases significantly when casts obstruct the majority of the airway, with most deaths attributable to central airway obstruction (31). Predictors of poor prognosis include: delayed diagnosis, large cast burden requiring repeated bronchoscopic interventions, involvement of central airways, presence of underlying PLE, and failure to respond to first-line therapies (31, 34, 57).

## 8 Limitations and future directions

### 8.1 Limitations of current evidence

Several important limitations must be acknowledged when interpreting the current evidence on pediatric PB. First, the vast majority of published data consist of Level IV–V evidence, including retrospective cohort studies, case series, and single case reports. There are no large-scale randomized controlled trials evaluating specific PB treatments, and the small sample sizes of most studies limit the generalizability of findings. Second, significant heterogeneity exists across studies in terms of patient populations, definitions of PB, treatment protocols, and outcome measures, making cross-study comparisons challenging. Third, publication bias likely favors the reporting of severe or successfully treated cases, potentially overestimating both disease severity and treatment efficacy. Fourth, many studies lack long-term follow-up data, making it difficult to assess the durability of treatment responses and the true natural history of the disease.

### 8.2 Future research directions

Several areas warrant further investigation: (1) Large, multicenter prospective registries are needed to better characterize the epidemiology, clinical phenotypes, and natural history of pediatric PB across diverse populations. (2) Randomized controlled trials should be conducted to evaluate the efficacy and safety of specific interventions, including nebulized tPA, biologic agents, and lymphatic embolization, with standardized dosing protocols and outcome measures. (3) Biomarker studies may help identify predictors of treatment response and recurrence, enabling more personalized management strategies. (4) The development and validation of a unified classification system that integrates histopathological, imaging, and clinical data would improve diagnostic consistency and guide treatment selection. (5) Investigations into the basic mechanisms of mucin cross-linking, lymphatic dysfunction, and ET-mediated cast formation may reveal novel therapeutic targets. (6) The role of emerging biologic therapies, including anti-IL-5 and anti-IL-4/IL-13 agents, should be evaluated in well-designed prospective studies with adequate follow-up.

## 9 Summary

Plastic bronchitis is a potentially life-threatening airway disorder characterized by the formation of branching endobronchial casts. This literature review has synthesized the current evidence across the full spectrum of PB, from etiology through management and outcomes, highlighting several key points for clinical practice.

**Key clinical take-home messages** include: (1) A high index of suspicion is essential for early diagnosis, particularly in children with a history of Fontan surgery or severe respiratory infections. (2) Flexible bronchoscopy remains the cornerstone for both diagnosis and initial treatment, with serial procedures often required for adequate cast clearance. (3) The management approach should be differentiated based on the underlying etiology: cardiac-associated PB benefits from lymphatic assessment and targeted embolization, while infection-associated PB requires prompt antimicrobial and anti-inflammatory therapy. (4) DCMRL has transformed the management of post-Fontan PB by enabling visualization and intervention on pathologic lymphatic channels. (5) Emerging therapies, including nebulized tPA and biologic agents, offer promise for refractory cases but require further study.

Areas of ongoing controversy include the optimal dosing and duration of heparin and tPA therapy, the role of biologic agents as first-line vs. rescue therapy, the indications and timing of lymphatic embolization, and the relative contributions of inflammatory vs. lymphatic mechanisms in mixed-etiology cases.

The field of pediatric PB management is evolving rapidly, with recent systematic reviews, prospective clinical trials, and multicenter cohort studies providing an increasingly robust evidence base. However, the rarity of the condition and the predominance of low-level evidence underscore the need for collaborative, multicenter research efforts to standardize diagnostic criteria, evaluate treatments, and ultimately improve outcomes for affected children.
